# Health-related quality of life in relapsing remitting multiple sclerosis patients during treatment with glatiramer acetate: a prospective, observational, international, multi-centre study

**DOI:** 10.1186/1477-7525-8-133

**Published:** 2010-11-15

**Authors:** Peter J Jongen, Dirk Lehnick, Evert Sanders, Pierette Seeldrayers, Sten Fredrikson, Magnus Andersson, Joachim Speck

**Affiliations:** 1 MS4 Research Institute, Ubbergseweg 34, 6522 KJ Nijmegen, the Netherlands; 2 STATPROC, Hohentengen am Hochrhein, Germany; 3 Amphia Hospital, Breda, the Netherlands; 4 Hospital Civil de Charleroi, Charleroi, Belgium; 5 Division of Neurology Huddinge, Department of Clinical Neuroscience, Karolinska Institutet, Stockholm, Sweden; 6 Karolinska Universitetssjukhuset i Solna, Solna, Sweden; 7 Nuvisan GmbH, Neu-Ulm, Germany

## Abstract

**Background:**

Glatiramer acetate (GA) and interferon-beta (INFb) are first-line disease modifying drugs for relapsing remitting multiple sclerosis (RRMS). Treatment with INFb is associated with a significant increase in health-related quality of life (HR-QoL) in the first 12 months. It is not known whether HR-QoL increases during treatment with GA.

**Methods:**

197 RRMS patients, 106 without and 91 with prior immunomodulation/immunosuppression, were studied for HR-QoL (Leeds Multiple Sclerosis-QoL [LMS-QoL] scale, score range 0 - 32), fatigue (Fatigue Impact Scale [FIS]) and depressed mood (Beck Depression Inventory-Short Form [BDI-SF]) at baseline and 6 and 12 months after start of GA treatment.

**Results:**

At 6 and 12 months mean LMS-QoL scores were significantly increased in the treatment-naive patient group (p < 0.001), not in the pre-treated group. At month 12 43% of treatment-naïve patients had improved HR-QoL (increase LMS-QoL score 3 or more points) (p < 0.001). Likewise, mean FIS scores were decreased at months 6 and 12 in the treatment-naïve group (p < 0.01), not in the pre-treated group. In both groups mean BDI-SF scores did not change. No demographic or clinical baseline factor was predictive of HR-QoL increase. HR-QoL changes were zero to negative for patients who had discontinued GA before month 12 (28.4% of patients).

**Conclusions:**

In RRMS patients without prior immunomodulation/immunosuppression treatment with GA was associated with an increase in HR-QoL in the first 6 months, that was sustained at 12 months. In 4 out of 10 patients HR-QoL improved. Increase in HR-QoL was associated with decrease in fatigue.

## Introduction

The efficacy of the disease modifying drug (DMD) glatiramer acetate (GA) in reducing relapses in relapsing remitting multiple sclerosis (RRMS) has been demonstrated in randomized placebo-controlled trials [1,2]. Studies on the effectiveness of GA treatment in daily neurological practice have concentrated on relapses, disability, fatigue [3-5], work absenteeism [4] and cost-effectiveness [6,7].

Health-related quality of life (HR-QoL) is an overall measure of effectiveness from a patient's perspective and is becoming increasingly important in the assessment of therapies in chronic disorders. It has been demonstrated that in MS patients treatment with the DMD interferon-beta (INFb) is associated with an increase in HR-QoL [8,9]. Data on HR-QoL during GA treatment are scarce and not informative [8,10].

Fatigue and depression are primary determinants of impaired HR-QoL in MS [11]. Fatigue is reported by over 80% of patients [12] and often interferes with social or occupational activities [13]. It has been suggested that GA may improve MS-related fatigue [3,4]. Depressive symptoms are also frequent in MS, the life-time prevalence of major depressive disorders being 22% [14].

To investigate the overall benefit RRMS patients perceive from GA treatment we performed a prospective, observational, international, multi-centre study in patients treated with GA in daily practice, focusing on HR-QoL, fatigue, and depression (FOCUS study). Two groups were studied, patients with prior immunomodulation/immunosuppression (pre-treated group) and patients without such treatment (treatment-naïve group) [15]. Here we report the results.

## Methods

### Study and study populations

Investigator-initiated, prospective, observational, international, multi-centre study. Patients were recruited in out-patient departments from general hospitals, academic hospitals and MS centers in the Netherlands, Sweden and Belgium. The protocol was submitted to the Independent Review Board (IRB), an approved ethical committee residing in Amsterdam, the Netherlands. The IRB concluded that, because of the observational design of the study, a review by an ethical committee was not required. The study did not qualify for being tested according to the Dutch Medical Research Involving Human Subjects Act of 1999 [16]. Patients signed an informed consent form before any study related procedure was performed. The study was carried out in compliance with the Helsinki Declaration. The study was funded by unrestricted grants from TEVA Pharma Netherlands, Sweden and Belgium.

Inclusion criteria: 1) 18 years or older, 2) Expanded Disability Status Scale (EDSS) score less than 5.5, and 3) relapse- and steroid-free for at least 30 days. Exclusion criteria: 1) hypersensitivity to GA or mannitol, 2) previous treatment with GA, and 3) pregnancy or lactation.

HR-QoL, fatigue, depression, disability and relapses were assessed at baseline and at 6 and 12 months. In case of a relapse a scheduled study visit was postponed until at least 30 days after recovery.

In 29 centers 42 investigators, members of the FOCUS study group, enrolled 106 treatment-naïve and 91 pre-treated patients (Table 1). One-hundred-thirty-five patients were included in the Netherlands, 42 in Sweden and 20 in Belgium.

**Table 1:** Demographic and neurological characteristics of patient groups.

		Total group	Treatment-naive group	Pre-treated group
		N = 197	N = 106	N = 91
Gender	Female (%)	151 (76.6)	81 (76.4)	70 (76.9)
	Male (%)	46 (23.4)	25 (23.6)	21 (23.1)
Age (years)	Mean (SD)	38.6 (9.6)	38.5 (9.9)	38.8 (9.2)
Disease duration (years)*	Mean (SD)	4.3 (4.7)	2.7 (4.0)	6.3 (4.8)
GNDS score*	Mean (SD)	10.34 (5.56)	8.75 (4.60)	12.19 (6.01)
EDSS score	Mean (SD)	2.5 (1.42)	2.2 (1.14)	2.9 (1.65)
ARR	Mean (SD)	1.15 (0.62)	1.13 (0.50)	1.18 (0.73)

SD, standard deviation; GNDS, Guy's Neurological Disability Scale; EDSS, Expanded Disability Status Scale; ARR, annualized relapse rate. *, P < 0.05 for differences between treatment-naïve and pre-treated groups.

### Outcome measures and assessments

HR-QoL was assessed by the Leeds Multiple Sclerosis Quality of Life (LMS-QoL) scale. In 2000 the LMS-QoL scale was developed in a community-based population of people with MS [17]. The scale has been subjected to standard psychometric testing of validity and reliability and to Rasch analysis. It was found to fit the Rasch model and had a close association to well being, with good internal consistency and test-retest reliability. It is a self-assessment scale that consists of eight questions, examining MS-related aspects of QoL over the past month. The LMS-QoL is easy to use and practical to administer in a clinic setting or as postal questionnaire [17]. Answers are rated on a 5-point scale from 0 to 4. The resulting score ranges from 8 to 32, with higher scores reflecting higher levels of well being. It returned normally distributed scores when tested in a population of MS patients [18], without significant floor or ceiling effects. In a study of MS patients with acute relapses, it was found to be responsive to change with higher effect sizes than the Euro-Qol instrument and all of the subscales of the MSQoL-54 [19], except measuring social function [20]. In a separate study it showed significant correlation with a detailed impact diary [21].

Fatigue was assessed by the Fatigue Impact Scale (FIS) [22]. The FIS is a validated questionnaire examining perception of fatigue over the past month and consists of a total of 40 questions in 3 domains: cognitive, physical and social. Answers are rated on a 5-point scale (0 to 4). Total FIS score ranges from 0 to 160 and higher total score indicates more experienced fatigue. The FIS is widely accepted for assessment of fatigue in patients with MS [3,23].

Depression was assessed by the Beck Depression Inventory-Short Form (BDI-SF). The BDI-SF is a short validated questionnaire of 13 questions [24,25]. Answers are rated on a 4-point scale (0 to 3). Total score ranges from 0 to 39 and higher scores indicate more depressed mood.

Disability was assessed by the Guy's Neurological Disability Scale (GNDS), a validated questionnaire measuring 12 domains of disability [26]. GNDS score ranges from 0 to 60, where higher scores indicate increased disability. The GNDS has a good correlation to EDSS [27]. EDSS was assessed at baseline.

LMS-QoL, FIS and BDI-SF were completed by patients at home within 7 days before study visit or at the clinic just prior to the visit. Investigators completed the GNDS by interview during visits.

### Statistical analyses

Changes from baseline were calculated for the total group, treatment-naïve group, and pre-treated group. Given the statistically significant differences in baseline characteristics for pre-treated and treatment-naïve patient groups, differences during treatment were not statistically tested.

Changes are given as mean value ± standard deviation (SD) with 95% confidence interval (CI) for the mean change. If a 95% CI for the mean change does not include zero the change may be considered significant in an exploratory manner since no adjustments for multiple testing were applied. When a patient terminated GA treatment before month 12 a final assessment occurred as for the next visit (month 6 or 12) and Last Observation Carried Forward was applied. In a post-hoc analysis demographics and LMS-QoL scores were investigated separately for patients who continued vs. patients who discontinued GA.

In individual patients an increase in LMS-QoL score of 3 or more points was classified as improvement, decrease of 3 or more points as worsening. Within each group a Wilcoxon signed-rank test was performed to assess whether there was a tendency different from 'no change'.

Logistic regression analysis was applied to determine whether predefined factors - age, gender, disease duration, prior treatment and baseline scores for FIS, BDI-SF, GNDS and EDSS - were predictive of LMS-QoL change.

## Results

### Baseline and discontinuation data

Most pre-treated patients (94.5%) had received immunomodulation, mainly IFNb. Five patients (5.5%) had received mitoxantrone or methotrexate. The pre-treated group had higher mean values for disease duration and disability than the treatment-naïve group (Table 1).

One-hundred-forty-one patients (71.6%) completed 12 months treatment: 83 treatment-naïve (78.3%) and 58 pre-treated (63.7%). Reasons for discontinuation of GA treatment were side effects (n = 41), lack of effectiveness (n = 5), wish for pregnancy (n = 2), withdrawal of consent (n = 1), death (n = 1) or unknown (n = 6). All patients who completed 12 months GA treatment also completed study participation.

### HR-QoL

Values for LMS-QoL at baseline, month 6 and month 12 and changes from baseline are given in table 2.

**Table 2:** LMS-QoL values and changes from baseline.

Time points and periods	Parameters	Total group	Treatment-naive group	Pre-treated group
Baseline (B)	Mean (SD)	19.51 (4.19)	19.54 (4.26)	19.47 (4.12)
	Median	19.00	20.00	19.00
	95% CI (mean)	18.92; 20.10	18.72; 20.36	18.61; 20.33
Month 6 (M6)	Mean (SD)	20.95 (4.63)	21.35 (4.63)	20.48 (4.61)
	Median	21.00	22.00	21.00
	95% CI (mean)	20.26; 21.65	20.40; 22.31	19.45; 21.51
Month 12 (M12)	Mean (SD)	20.94 (4.69)	21.73 (4.76)	19.98 (4.43)
	Median	21.00	21.50	20.00
	95% CI (mean)	20.25; 21.63	20.78; 22.69	19.00; 20.96
Change B to M6	Mean (SD)	1.41 (3.97)*	1.90 (4.08)*	0.82 (3.77)
	Median	1.00	1.00	1.00
	95% CI (mean)	0.81; 2.00	1.06; 2.74	-0.02; 1.67
Change B to M12	Mean (SD)	1.33 (4.24)*	2.10 (4.56)*	0.40 (3.64)
	Median	1.00	1.00	1.00
	95% CI (mean)	0.70; 1.96	1.19; 3.02	-0.41; 1.20

SD, standard deviation; CI, confidence interval; *, p < 0.001. Differences between groups were not statistically tested.

Both at 6 and 12 months mean LMS-QoL scores were significantly increased in the treatment-naive and the total group, not in the pre-treated group (Table 2).

At month 6 41 patients (44.1%) in the treatment-naïve group had improved HR-QoL, whereas HR-QoL was unchanged in 43 (44.2%) and had worsened in 9 (9.7%) (p < 0.001). For pre-treated patients there was no central tendency different from 'no change' (p = 0.23). In the total group 61 patients (35.5%) had improved, 89 (51.7%) were unchanged and 22 (12.8%) had worsened (p < 0.001).

At 12 months results were similar. In the treatment-naïve group 42 patients (42.9%) had improved HR-QoL, while 44 (44.9%) had unchanged and 12 (12.2%) worsened HR-QoL (p < 0.001). No such tendency was seen in pre-treated patients (p = 0.13). In the total group 64 patients (35.8%) had improved, 90 (50.3%) were unchanged, and 25 (14.0%) had worsened (p < 0.001).

A post hoc analysis was performed comparing patients who had completed 12 months treatment (71.6%) to those who had discontinued treatment (28.4%). Non-completion was more frequent in pre-treated (36.3%) than in treatment-naïve patients (22.0%). In both groups LMS-QoL changes were about 2 points more advantageous for completers than for non-completers. Mean changes were close to zero or slightly negative for non-completers, while results for completers were even slightly better than unstratified values (Table 3).

**Table 3:** LMS-QoL values for completers vs. non-completers and percentages of patients with improved, unchanged and worsened HR-QoL at month 12.

LMS-QoL	Parameters	Total group completers	Total group non-completers	Treatment-naive completers	Treatment-naive non-completers
Baseline (B)	Mean (SD)	19.84 (4.15)	18.66 (4.20)	19.59 (4.23)	19.35 (4.49)
	Median	20.00	19.00	19.00	20.00
	95% CI (mean)	19.15; 20.54	17.54; 19.79	18.67; 20.51	17.41; 21.29
Change B to M12	Mean (SD)	1.76 (4.25)*	-0.21 (3.91)	2.52 (4.72)*	-0.06 (2.91)
	Median	1.00	0.00	2.00	0.00
	95% CI (mean)	1.05; 2.47	-1.47; 1.06	1.49; 3.56	-1.61; 1.49
Improved M12	n (%)	59 (42.1)	5 (12.8)	40 (48.8)	2 (12.5)
Unchanged	n (%)	65 (46.4)	25 (64.1)	34 (41.5)	10 (62.5)
Worsened	n (%)	16 (11.4)	9 (23.1)	8 (9.8)	4 (25.0)

*, p < 0.001.

Multiple regression models not including baseline HR-QoL as additional factor explained less than 15% of variability of HR-QoL changes. The improved performance of models including baseline HR-QoL - still not explaining more than 40% of variability - relates to the fact that patients with low baseline HR-QoL have a better chance for improvement.

### Fatigue and depression

Table 4 shows FIS values in treatment-naïve, pre-treated and total patient groups at baseline, month 6 and month 12, and changes from baseline.

**Table 4:** FIS values and differences from baseline.

Time points and periods	Parameters	Total Group	Treatment-naive group	Pre-treated group
Baseline (B)	Mean (SD)	60.53 (34.24)	56.25 (32.65)	65.51 (35.54)
	Median	59.00	55.00	70.00
	95% CI (mean)	55.72; 65.34	49.97; 62.54	58.10; 72.91
Month 6 (M6)	Mean (SD)	51.15 (35.35)	45.68 (33.33)	57.79 (36.76)
	Median	44.50	40.00	50.00
	95% CI (mean)	45.83; 56.47	38.81; 52.54	49.36; 65.83
Month 12 (M12)	Mean (SD)	54.07 (35.18)	47.97 (33.78)	61.46 (35.62)
	Median	49.00	43.50	63.00
	95% CI (mean)	48.88; 59.26	41.20; 54.74	53.58; 69.33
Change B to M6	Mean (SD)	-9.17 (27.08)***	-11.33 (27.67)***	-6.62 (26.32)*
	Median	-6.00	-7.00	-3.00
	95% CI (mean)	-13.24; -5.09	-17.03; -5.64	-12.51; -0.73
Change B to M12	Mean (SD)	-6.04 (29.04)**	-8.50 (29.35)**	-3.06 (28.57)
	Median	-5.00	-8.00	-3.00
	95% CI (mean)	-10.32; -1.76	-14.38; -2.62	-9.38; 3.25

SD, standard deviation; CI, confidence interval; ***, p < 0.001; **, p < 0.01; *, p < 0.05. Differences between groups were not statistically tested.

At month 6 mean total FIS score had decreased in the treatment-naïve, pre-treated, and total groups. At month 12, compared to baseline, mean total FIS score had decreased in the treatment-naïve group and the total group, not in the pre-treated group.

At baseline mean BDI-SF score in the treatment-naive group was 5.81 (SD 4.03), in the pre-treated group 7.47 (SD 5.29), and in the total group 6.58 (SD 4.72). There were no statistically significant changes at month 6 or 12 in any group.

### Disability and relapses

At 6 months mean GNDS score in the treatment-naïve group (7.40; SD 4.80) and in the total group (9.57, SD 6.00) were lower than at baseline (p = 0.001 and p = 0.01, resp.). Other changes were not statistically significant.

Annualized relapse rate (ARR) over the study period was 0.69 (SD 0.96) in the treatment-naïve group, 1.03 (SD 1.22) in the pre-treated group, and 0.84 (SD 1.09) in the total group.

## Discussion

We observed a mean increase in HR-QoL in treatment-naïve RRMS patients 6 months after start of GA treatment, that was sustained at 12 months. Both mean and median HR-QoL values were higher than at baseline, suggesting that the increase was a group phenomenon, rather than caused by individual outliers. HR-QoL increase was paralleled by a decrease in experienced fatigue, whereas levels of depressed mood remained unchanged.

In a single-centre study Lily et al. assessed HR-Qol in 210 RRMS patients before and during treatment with disease modifying drugs (DMD) - intramuscular (IM) INFb-1a, subcutaneous INFb-1a, subcutaneous INFb-1b, GA - and found that DMD treatment was associated with an increase in HR-QoL [8]: Mean HR-QoL score increased significantly at one month, further increasing to a peak at nine months, and remaining significantly elevated at three years. The small number - eight - of GA-treated patients in their study did not allow for conclusions with respect to GA. Our data show that increase in HR-QoL is also specifically associated with GA treatment. Moreover, our finding that HR-QoL was increased at 6 months and remained elevated at 12 months is in agreement with the pattern described by Lily et al. [8].

It has been known that the relationship between disability and HR-QoL in MS is complex [28]. In treatment-naive patients we observed an improvement of mean disability score (GNDS) at 6 months, but not at 12 months. In their study Lily et al. found no significant change in median disability (EDSS) throughout the three year study period [8]. Likewise, we found that disability remained unchanged in RRMS patients during two years of treatment with IM INFb-1a, whereas mean physical and mental HR-QoL scores (MS54-QoL) improved significantly [9]. In fact, as prevention of disability progression is a primary goal of DMD treatment, staying clinically stable is per se a beneficial phenomenon. Patients may experience stability as a relative improvement, that positively affects HR-QoL.

No baseline characteristic was predictive of HR-QoL increase, except for low HR-QoL. In contrast, in a study in IM INFb-1a-treated RRMS patients it was found that increase in HR-QoL was associated with young age and low baseline disability [9]. Lily et al. performed multiple regression analysis of baseline characteristics against change in HR-QoL score over the first two years of treatment with DMD [8]. There was no relationship with age, disease duration, disability or number of relapses, and - similar to out finding - the only significant variable predicting a good QoL response was low QoL [8]. In all, age, disease duration, disability, fatigue or depressed mood are characteristics that are not helpful in selecting patients for GA treatment in terms of QoL improvement.

After 12 months treatment-naive patients showed a mean increase in LMS-QoL score of about 11%. It is not known what change in HR-QoL is relevant for individual MS patients. In general, changes of 10% to 15% in clinical outcomes may be considered meaningful. We choose a minimum of 15% of the mean baseline LMS-QoL score in the total population (19.51), being 3 points, for an individual change to be qualified as improvement or worsening. Thus, after one year 4 out of 10 treatment-naive patients had an improved HR-QoL and only 1 out of 10 a worsened HR-QoL. In patients who had completed 12 months treatment changes were even more advantageous, whereas for non-completers mean changes were close to zero or even negative. As most patients who discontinued GA did so because of side effects (73%), in non-completers the negative impact of side effects on HR-QoL may have outweighed GA's positive effect [29]. Moreover, treatment discontinuation could have prevented the full development of GA's beneficial effect, as it is known that relapse reduction occurs not sooner than after 6 months.

Interestingly, in patients with prior immunomodulation/immunosuppression mean HR-QoL did not change significantly. This could be explained by several factors. First, overrepresentation in this group of patients with more active inflammation, as it is likely that a substantial number of patients had stopped previous treatment for reason of incomplete response. In spite of prior treatment, pre-study relapse rate in this group was similar to that in naive patients and on-study relapse rate seemed even higher. Second, the placebo effect was perhaps more modest in this group, as patients had had the disappointing experience of treatment failure, although we think it unlikely that at 12 months a significant placebo effect was still at work in either group. Third, unchanged HR-QoL may relate to the higher rate of non-completers in this group (36%).

At 12 months treatment-naive patients showed a significant decrease in fatigue. Decreased fatigue in MS patients during the first year of GA treatment has been reported by others [3,4]. Our observation that a decrease in fatigue was associated with an increase in HR-QoL suggests that a decline in experienced fatigue contributes to HR-QoL improvement.

Our study does not inform on HR-QoL beyond the first year of GA treatment. Comparison with the report by Lily et al. shows a similar pattern of early increase in the first 6 months, that is sustained at 12 months, which suggests that this early gain in HR-QoL may be sustained further [8]. Yet, future studies are needed to establish the long-term change in HR-QoL during GA treatment.

In order to weigh expected benefits against risks patients need quantified information on effectiveness before deciding to start DMD treatment. Randomized controlled trials (RCTs) investigate efficacy and mostly do not include overall or patient-centred measures. Moreover, RCT data are obtained in settings not representative of daily practice and thus difficult to generalize. Observational data obtained in 'real-life' are more informative on a patient's chance to reach a pre-defined therapeutic goal. As our patients were treated in daily care settings, this study's results may be considered representative of GA treatment in real life.

## Conclusion

In treatment-naïve RRMS patients treatment with GA was associated with an increase in HR-QoL in the first 6 months, that was sustained at 12 months. Moreover, increase in HR-QoL was associated with decrease in fatigue. Our finding in this group that 4 out of 10 patients had improved HR-QoL 12 months after start of GA treatment suggests that clinically active RRMS patients without prior immunomodulation/immunosuppression have approximately 40% chance of improved HR-QoL one year after start of GA treatment. This information may be helpful to neurologists and patients when considering immunomodulating treatment.

## Competing interests

Dr. Jongen has received honoraria from Sanofi-Aventis, Teva, Merck-Serono, Novartis, Bayer-Schering, Biogen-Idec and Allergan for activities as speaker, advisory committee member, research support, or travel grants for conferences. Prof Fredrikson has received honoraria for lectures and educational activities from Sanofi-Aventis, Teva, Bayer-Schering, Biogen-Idec and Merck-Serono.

## Authors' contributions

PJJ initiated the study, contributed to conception and design of the study, acquisition of data, analysis of data and interpretation of data, drafted the manuscript, and has given final approval of the version to be published. DL contributed to conception and design of the study, performed data analysis, contributed to interpretation of data, drafted the manuscript, and has given final approval of the version to be published. ES contributed to conception and design of the study, acquisition of data, interpretation of data, revised the manuscript critically for important intellectual content, and has given final approval of the version to be published. PS contributed to conception and design of the study, acquisition of data, interpretation of data, revised the manuscript critically for important intellectual content, and has given final approval of the version to be published. SF contributed to conception and design of the study, acquisition of data, interpretation of data, revised the manuscript critically for important intellectual content, and has given final approval of the version to be published. MA contributed to conception and design of the study, acquisition of data, interpretation of data, revised the manuscript critically for important intellectual content, and has given final approval of the version to be published. JS performed data analysis, contributed to interpretation of data, revised the manuscript critically for important intellectual content, and has given final approval of the version to be published. FOCUS study group members all contributed to acquisition of data.

## Authors' information

Peter Joseph Jongen is neurologist and founding director of the MS4 Research Institute, Nijmegen, the Netherlands. He has been involved in MS clinical research and patient care for more than 15 years. He is member of the International Medical and Scientific Board of the Multiple Sclerosis International Federation (MSIF), co-founder and former director of the MS Centre Nijmegen, former council member of the European Committee for Treatment and Research in Multiple Sclerosis (ECTRIMS), and author of over 90 peer-reviewed scientific articles. The MS4 Research Institute conceives, performs and coordinates scientific research on the therapeutic value of treatments in MS.

Sten Fredrikson is Professor of Neurology at Karolinska Institutet, Stockholm, Sweden. He has been involved in MS research for more than 25 years and has published more than 150 peer-reviewed scientific articles and many other review articles and book chapters.
